# Assessing the Impacts of Meteorological Factors on COVID-19 Pandemic Using Generalized Estimating Equations

**DOI:** 10.3389/fpubh.2022.920312

**Published:** 2022-07-01

**Authors:** Shengnan Lin, Jia Rui, Fang Xie, Meirong Zhan, Qiuping Chen, Bin Zhao, Yuanzhao Zhu, Zhuoyang Li, Bin Deng, Shanshan Yu, An Li, Yanshu Ke, Wenwen Zeng, Yanhua Su, Yi-Chen Chiang, Tianmu Chen

**Affiliations:** ^1^School of Public Health, Xiamen University, Xiamen, China; ^2^Cirad, UMR 17, Intertryp, Université de Montpellier, Montpellier, France; ^3^Fujian Provincial Center for Disease Control and Prevention, Fuzhou, China; ^4^Clinical Medical Laboratory, Xiang'an Hospital of Xiamen University, Xiamen, China

**Keywords:** COVID-19, meteorological factors, transmissibility, generalized estimating equations, lagged effect

## Abstract

**Background:**

Meteorological factors have been proven to affect pathogens; both the transmission routes and other intermediate. Many studies have worked on assessing how those meteorological factors would influence the transmissibility of COVID-19. In this study, we used generalized estimating equations to evaluate the impact of meteorological factors on Coronavirus disease 2019 (COVID-19) by using three outcome variables, which are transmissibility, incidence rate, and the number of reported cases.

**Methods:**

In this study, the data on the daily number of new cases and deaths of COVID-19 in 30 provinces and cities nationwide were obtained from the provincial and municipal health committees, while the data from 682 conventional weather stations in the selected provinces and cities were obtained from the website of the China Meteorological Administration. We built a Susceptible-Exposed-Symptomatic-Asymptomatic-Recovered/Removed (SEIAR) model to fit the data, then we calculated the transmissibility of COVID-19 using an indicator of the effective reproduction number (*R*_*eff*_). To quantify the different impacts of meteorological factors on several outcome variables including transmissibility, incidence rate, and the number of reported cases of COVID-19, we collected panel data and used generalized estimating equations. We also explored whether there is a lag effect and the different times of meteorological factors on the three outcome variables.

**Results:**

Precipitation and wind speed had a negative effect on transmissibility, incidence rate, and the number of reported cases, while humidity had a positive effect on them. The higher the temperature, the lower the transmissibility. The temperature had a lag effect on the incidence rate, while the remaining five meteorological factors had immediate and lag effects on the incidence rate and the number of reported cases.

**Conclusion:**

Meteorological factors had similar effects on incidence rate and number of reported cases, but different effects on transmissibility. Temperature, relative humidity, precipitation, sunshine hours, and wind speed had immediate and lag effects on transmissibility, but with different lag times. An increase in temperature may first cause a decrease in virus transmissibility and then lead to a decrease in incidence rate. Also, the mechanism of the role of meteorological factors in the process of transmissibility to incidence rate needs to be further explored.

## Introduction

As the country with the third-largest land area range in the world, China has a wide range of climates from north to south. It was found that temperature could be the most important predictor of growth rate during the COVID-19 outbreak (1, 2). When the temperature increases, the basic reproduction number (*R*_0_) continues to decrease (3), as well as the mortality rate of moderate and severe patients (4, 5). In addition, air humidity is the main climatic factor influencing the development of the COVID-19 epidemic (6, 7). Some studies have shown a negative correlation between absolute humidity and the number of reported cases (8), while others have concluded that there is a positive correlation between the number of reported cases and relative humidity and absolute humidity (9). Studies have also shown that temperature and relative humidity are the main drivers of the COVID-19 epidemic, and they vary with season and geographic location (10). In summer, increased relative humidity and decreased maximum temperature promoted the spread of COVID-19 in inland cities, while decreased relative humidity favored the spread of COVID-19 in coastal cities (10). For relatively humid coastal cities, lower relative humidity and higher winter minimum temperatures promote the spread of COVID-19 (10). In addition, the effects of wind speed and precipitation on COVID-19 have shown different results in different studies (8, 11–14). As can be seen from the above studies, differences may exist when the outcome variables of the study are incidence rate, fatality rate, or number of cases. Also, the outcome variable was mostly single across studies, but differences in socioeconomic background (2) and prevention and control measures (15) in the selected areas in different studies may affect the results. To quantify the differences in the effects of meteorological factors on different outcome variables, this study planned to analyze the effects of key meteorological factors on the transmissibility, incidence rate, and number of COVID-19 in the same study and to compare whether there were differences in their effects on different outcome variables.

In addition, lack of knowledge of meteorological data may also be a cause of discrepancies (16). In addition, meteorological factors are changing daily. If we can analyze their effects on COVID-19 with serial data over time, we can reveal the effects of meteorological factors more accurately (17). In the study of the relationship between key meteorological factors and hand-foot-and-mouth disease: it was proposed that the effective reproduction number (*R*_*eff*_) may be an intermediate link between meteorological factors and incidence rate (18), that the effect of meteorological factors on transmissibility may precede the number of reported cases. Based on this, the study will explore the differences in the effects of key meteorological factors on different outcome variables using panel data and further assess whether there is a lagged effect of meteorological factors on the three outcome variables and the lagged time differences.

## Methods

### Data Collection

In this study, the daily number of reported cases and deaths of COVID-19 was obtained from the provincial and municipal health commissions in China, and the meteorological data were obtained from the website of the China Meteorological Administration (http://www.cma.gov.cn/). Since only one cumulative confirmed case was found in Tibet, and meteorological data were not available for Hong Kong Special Administrative Region, Macau Special Administrative Region, and Taiwan Province, data from 682 conventional meteorological stations (Supplementary Figure 1) in 30 provinces, autonomous regions, and municipalities directly under the central government were finally used in this study. The meteorological data included temperature (°C), relative humidity (%), precipitation (mm), sunshine hours (h), air pressure (hPa), and wind speed (m/s) at each station. On December 7, 2019, the first confirmed case of the COVID-19 was reported in China, while on January 10, 2020, the case was first reported on the official website of the Wuhan Municipal Health Planning Commission. Considering the completeness and continuity of the data, the starting date of data collection was January 10, 2020. The first imported case in China was reported on February 26, 2020, and to control for the impact of the imported cases on our study, we collected national outbreak data before the end of February 26, 2020. We also included holiday as a categorical variable in the generalized estimation model to control for potential confounding effects. In China, there are traditional holidays, including the Spring Festival, in addition to Saturdays and Sundays, which are included in this study period. More importantly, due to the epidemic, the State Council extended the week-long Spring Festival holiday to February 2, 2020. Therefore, we defined each date during this study period based on the Chinese State Council's holiday notification (1 = holiday, 0 = working day). In addition, we collected data on population, birth rate, and death rate for each province and city.

### SEIAR Model Building

We built the Susceptible–Exposed–Symptomatic–Asymptomatic-Recovered/Removed (SEIAR) model based on previous researches of our group (19, 20). To implement the model, individuals were divided into the following five categories: susceptible (*S*), exposed (*E*), symptomatic (*I*), asymptomatic (*A*), and recovered/removed (*R*). Supplementary Figure 2 showed the framework of SEIAR model in detail.

The differential equations are:


$$\begin{array}{l} \frac{dS}{dt}=brN-\beta S\left(I+kA\right)-drS\\ \frac{dE}{dt}=\beta S\left(I+kA\right)-pw^{{}^{\prime }}E-\left(1-p\right)wE-drE\\ \frac{dI}{dt}=\left(1-p\right)wE-\gamma I-\left(dr+f\right)I\\ \frac{dA}{dt}=pw^{\prime }E-\gamma^{\prime }A-drA\\ \frac{dR}{dt}=\gamma I+\gamma^{\prime }A-drR \end{array}$$

### Parameter Estimation

Model parameter values and their sources were listed in Table 1. There were 10 parameters in this model, namely *br, dr*, β, κ, *p*, 1/ω, 1/ω', *f* , 1/γ and 1/γ'.

(1) The birth rate *br* and the mortality *dr* were derived from the statistical year books of corresponding regions.
(2) The actual report data of COVID-19 were fitted by SEIAR model to obtain the transmission relative rate β.
(3) In the early study, the transmissibility of asymptomatic infections was unable to determine, so this study was based on one article previously published by our team and set κ to 0.526 (19).
(4) In the early study, the proportion of asymptomatic infections was unable to determine, so this study was based on the articles previously published by the team and set *P* to 0.526 (19).
(5) Our study set the incubation period to 5 days (19, 22), and set the latent period to 5 days, that was ω = ω' = 0.2.
(6) The case fatality rate *f* of COVID-19 was calculated from the actual data, and its value was 0.02348.
(7) We set the infectious period to 6 days (1/γ = 1/γ' = 0.1667) (19, 22).

**Table 1:** Description and values of parameters in the susceptible–exposed–symptomatic–asymptomatic-recovered/removed (SEIAR) model.

**Parameter**	**Description**	**Unit**	**Value**	**Range**	**Method**
*br*	Birth rate	1	-	0–1	Regional statistical yearbook
*dr*	Mortality	1	-	0–1	Regional statistical yearbook
β	Transmission relative rate	Individuals^−1^·days^−1^	-	≥ 0	Curve fitting
κ	Relative transmissibility rate of asymptomatic to symptomatic individuals	1	0.5	–	(19, 21)
*P*	Proportion of the asymptomatic	1	0.5	–	(19)
Ω	Incubation relative rate	days^−1^	0.2	–	(19, 21, 22)
ω'	Latent relative rate	days^−1^	0.2	–	(19, 22)
γ	Recovered/removed rate of the infectious	days^−1^	0.1667	–	(19, 22)
*γ'*	Recovered/removed rate of the asymptomatic	days^−1^	0.1667	–	(19, 22)
*f*	Case fatality rate	1	0.02348	–	Analysis of data

### Indicator for Assessing Transmissibility

The *R*_0_ was usually used for quantitatively assessing the transmissibility (19, 23–27), but it described the natural transmissibility of the disease in an ideal state. In actual situations, the transmissibility of infectious diseases was generally measured by *R*_*eff*_. In this study, the formula for calculating *R*_*eff*_ is as follows:


$$R_{eff}=\beta S\left(\frac{1-p}{\gamma }+\frac{\kappa p}{\gamma^{\prime }}\right)$$

### Model Fitting and Statistical Analysis

This study used Berkeley Madonna 8.3.18 software for data fitting (developed by Robert Macey and George Oster of the University of California, Berkeley, Copyright 1993–2001 Robert I. Macey and George F. Oster), and adopted Fourth-order Runge–Kutta method (22, 28–32). Then, we set the tolerance to .001 to solve the differential equation. We used coefficient of determination (*R*^2^) calculated by IBM SPSS 21.0 to evaluate the curve fitting.

We organized the data by Microsoft Office Excel 2010 and draw the spatial distribution map of 682 meteorological stations. We calculated the daily incidence rates, temperature (°C), relative humidity (%), precipitation (mm), sunshine hours (h), air pressure (hPa), and wind speed (m/s) by IBM SPSS 21.0, and the daily meteorological data adopted the arithmetic average of all stations in the province and city; GraphPad Prism 7.0 was used for charting the above indicators.

The daily meteorological data of each city were set as independent variables. The daily *R*_*eff*_ value, incidence rate and the number of reported cases in each province were set as dependent variables, respectively. We used the generalized estimation equation to evaluate the short-time effect of independent variables on the dependent variables by SAS 9.4 software.

The working correlation matrix was used to evaluate the correlation of each repeated measurements and provided effective variance estimation for parameter estimates (33, 34). That is the correlation between the daily measurements of the dependent variables. Taking the *R*_*eff*_ value as an example: we can calculate the correlation coefficient between the *R*_*eff*_ value of the *i*^*th*^ day and the other *R*_*eff*_ value of the (*i*+1)^*th*^ day by working related matrix, to evaluate the correlation mentioned above.

This working correlation matrix include four different methods: (1) Autoregressive (1): AR (1): the correlation was related to the number of measurement intervals. The correlation was weak when the measurement intervals were far apart; (2) Exchangeable (EXCH) referred to the same correlation between any two measurements; (3) Unstructured (UN) referred to the off-diagonal data in a matrix formed by repeated measurements differing with each other; and (4) Independent (IND): it meant that there was no correlation between dependent variables of repeated measurement. In matrix selection, data types and generalized estimation equations fitting criterion quasi-likelihood under the independence model criterion (QIC) results were combined, in which the lower the QIC value, the better the model fit.

For example, take six repeated measurements as follows:


$$\text{AR}(\text{1}):\left(\begin{array}{c} \begin{array}{ccc} \text{1} & \rho & \rho^{\text{2}}\\ \rho & \text{1} & \rho \\ \rho^{\text{2}} & \rho & \text{1} \end{array}\begin{array}{ccc} \rho^{\text{3}} & \rho^{\text{4}} & \rho^{\text{5}}\\ \rho^{\text{2}} & \rho^{\text{3}} & \rho^{\text{4}}\\ \rho & \rho^{\text{2}} & \rho^{\text{3}} \end{array}\\ \begin{array}{ccc} \rho^{\text{3}} & \rho^{\text{2}} & \rho \\ \rho^{\text{4}} & \rho^{\text{3}} & \rho^{\text{2}}\\ \rho^{\text{4}} & \rho^{\text{4}} & \rho^{\text{3}} \end{array}\begin{array}{ccc} \text{1} & \rho & \rho^{\text{2}}\\ \rho & \text{1} & \rho \\ \rho^{\text{2}} & \rho & \text{1} \end{array} \end{array}\right)\text{EXCH}:\left(\begin{array}{c} \begin{array}{ccc} \text{1} & \rho & \rho \\ \rho & \text{1} & \rho \\ \rho & \rho & \text{1} \end{array}\begin{array}{ccc} \rho & \rho & \rho \\ \rho & \rho & \rho \\ \rho & \rho & \rho \end{array}\\ \begin{array}{ccc} \rho & \rho & \rho \\ \rho & \rho & \rho \\ \rho & \rho & \rho \end{array}\begin{array}{ccc} \text{1} & \rho & \rho \\ \rho & \text{1} & \rho \\ \rho & \rho & \text{1} \end{array} \end{array}\right)$$

$$\begin{array}{l} \text{UN}:\left(\begin{array}{c} \begin{array}{ccc} \text{1} & \rho_{\text{12}} & \rho_{\text{13}}\\ \rho_{\text{12}} & \text{1} & \rho_{\text{23}}\\ \rho_{\text{13}} & \rho_{\text{23}} & \text{1} \end{array}\begin{array}{ccc} \rho_{\text{14}} & \rho_{\text{15}} & \rho_{\text{16}}\\ \rho_{\text{24}} & \rho_{\text{25}} & \rho_{\text{26}}\\ \rho_{\text{34}} & \rho_{\text{35}} & \rho_{\text{36}} \end{array}\begin{array}{ccc} \rho_{\text{14}} & \rho_{\text{24}} & \rho_{\text{34}}\\ \rho_{\text{15}} & \rho_{\text{25}} & \rho_{\text{35}}\\ \rho_{\text{16}} & \rho_{\text{26}} & \rho_{\text{36}} \end{array}\begin{array}{ccc} \text{1} & \rho_{\text{45}} & \rho_{\text{46}}\\ \rho_{\text{45}} & \text{1} & \rho_{\text{56}}\\ \rho_{\text{46}} & \rho_{\text{56}} & \text{1} \end{array} \end{array}\right)\\ \text{ IND}:\left(\begin{array}{c} \begin{array}{ccc} \text{1} & \text{0} & \text{0}\\ \text{0} & \text{1} & \text{0}\\ \text{0} & \text{0} & \text{1} \end{array}\begin{array}{ccc} \text{0} & \text{0} & \text{0}\\ \text{0} & \text{0} & \text{0}\\ \text{0} & \text{0} & \text{0} \end{array}\begin{array}{ccc} \text{0} & \text{0} & \text{0}\\ \text{0} & \text{0} & \text{0}\\ \text{0} & \text{0} & \text{0} \end{array}\begin{array}{ccc} \text{1} & \text{0} & \text{0}\\ \text{0} & \text{1} & \text{0}\\ \text{0} & \text{0} & \text{1} \end{array} \end{array}\right) \end{array}$$

During the data analysis, it was necessary to create different databases to explore both immediate and lag effects. First, we needed to create a database to explore immediate effects; second, the dependent variable data for 1 day should be dropped and the dependent variable foe the next day corresponds to the meteorological factor values for the first day. By analogy, a database exploring lagged effects was established. In this study, model 1 is a model of the fitness effect, model 2 is a model of the 1-day lag effect, model 3 is a model of the 2-day lag effect, model 4 is a model of the 3-day lag effect model, model 5 is a model of the 4-day lag effect model, and model 6 is a model of the 5-day lag effect model.

## Results

### Description of the Changing Trend of Meteorological Factors at Provincial Level

**Figure 1:**
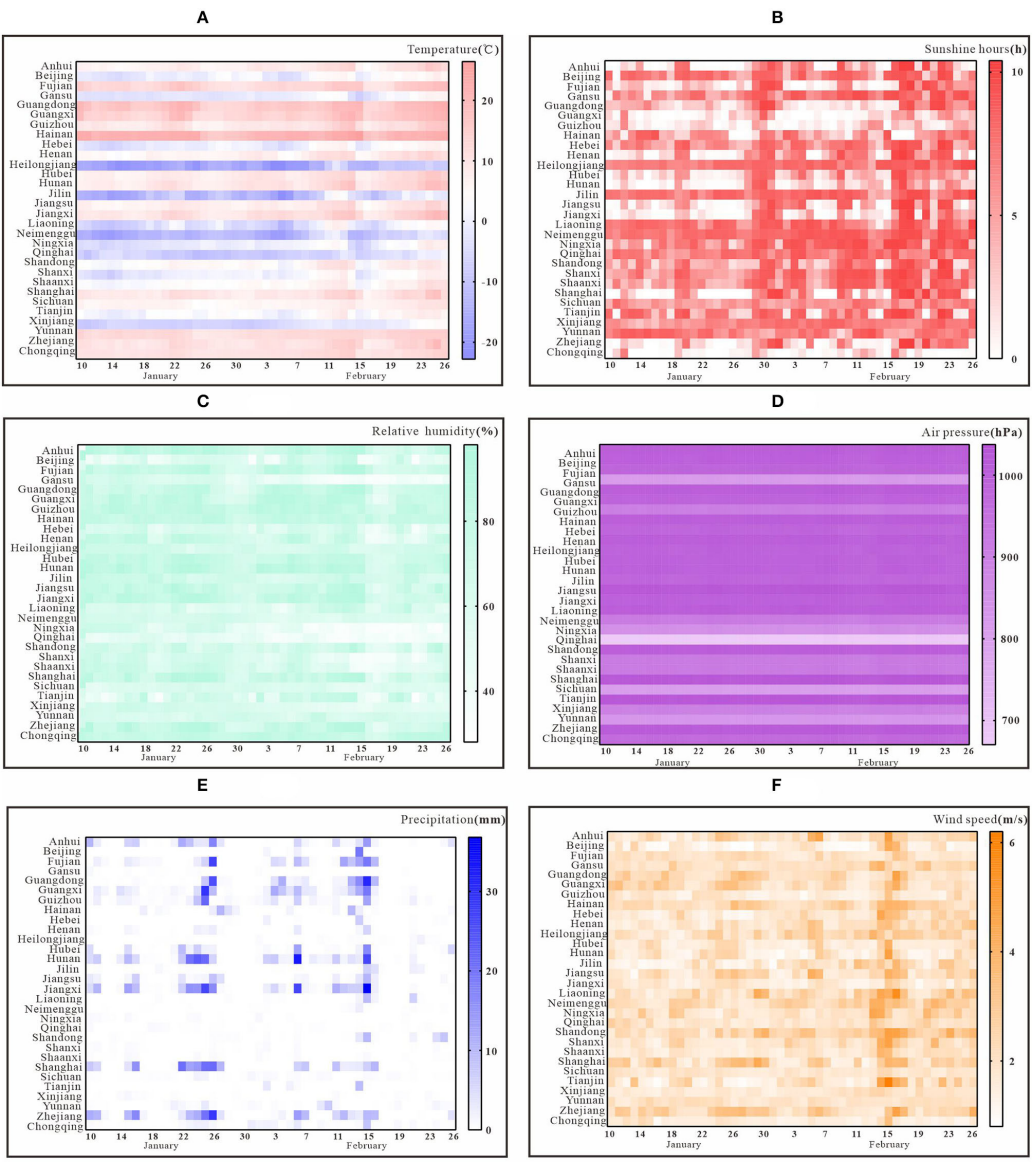
Change in trend of key meteorological factors in 30 provinces and cities. **(A)** daily average temperature; **(B)** relative humidity; **(C)** precipitation; **(D)** sunshine hours; **(E)** air pressure; **(F)** wind speed.

The spatial distribution map shows that 682 weather stations are distributed evenly in all provinces and cities. Figure 1A shows the trend of daily average temperature in each province and city. The trend of variation among provinces and cities is obvious, among which Heilongjiang Province, Jilin Province, and Inner Mongolia Autonomous have lower average daily temperature, while Hainan Province has higher average daily temperatures. The trend of daily average relative humidity is shown in Figure 1B. The daily relative humidity exceeded 20% in all provinces and cities and showed a fluctuating trend during the study period. Figure 1C shows the trend of daily average precipitation from January 10 to February 26, where most provinces and cities had low precipitation, with the least precipitation in Ningxia Province, Qinghai Province, Shaanxi Province, and Shanxi Province. Other provinces and cities show certain periodic changes in precipitation, with the most precipitation around January 22–26 and February 11–15.

Figure 1D shows the trend of daily average sunshine hours. The sunshine hours in Chongqing are relatively low. The sunshine hours vary somewhat among provinces and cities and show a clear trend of fluctuation over time. The trends of daily average air pressure are shown in Figure 1E. In general, all provinces and cities, except Qinghai Province, showed a relatively stable trend during the selected period. The trends of daily mean wind speed are shown in Figure 1F. In general, all provinces and cities have some differences and fluctuating trends in wind speed, with the peak wind speed occurring around February 15.

### The Trend of COVID-19 Transmissibility, Incidence Rate, and the Number of Reported Cases

**Figure 2:**
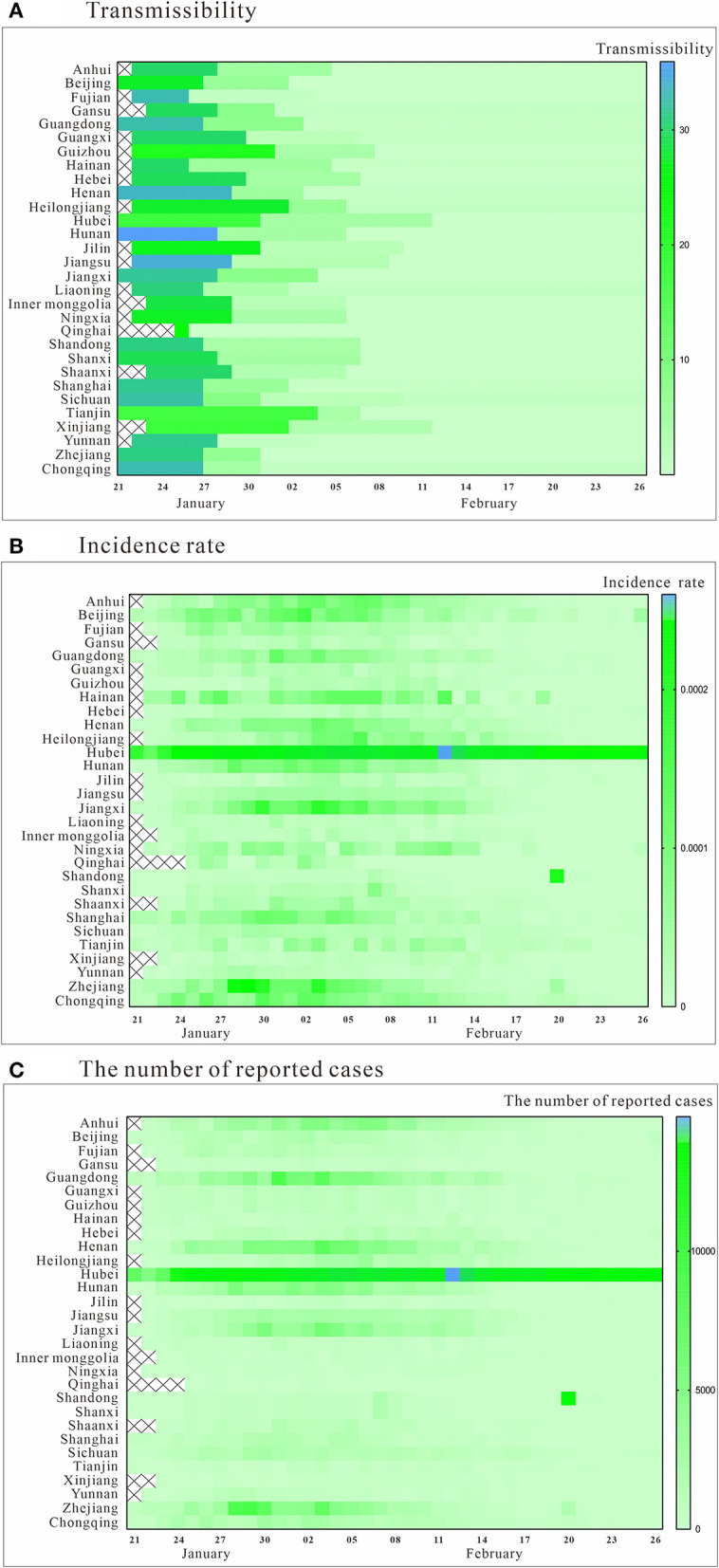
Trend of transmissibility, incidence rate, and the number of reported cases. **(A)** transmissibility; **(B)** incidence rate; **(C)** the number of reported cases.

The COVID-19 data from 30 Chinese provinces and cities were fitted using the SEIAR model. The results showed that the overall fit of the model was good (*P* < 0.05, Table 2). Figure 2A shows the trend of COVID-19 transmissibility in each province and city. It can be seen that the *R*_*eff*_ values of each province and city showed a decreasing trend over time. Among them, *R*_*eff*_ in Qinghai province first started to be below than 1 on January 26. After February 12, *R*_*eff*_ of all provinces and cities were <1.

**Table 2:** The results of goodness-of-fit in China.

**Provinces and cities**	***R^2^***	***P***
Beijing	0.732	<0.001
Tianjin	0.206	<0.05
Hebei province	0.435	<0.001
Shanxi province	0.545	<0.001
Inner monggolia autonomous region	0.282	<0.05
Liaoning province	0.212	<0.05
Jilin province	0.344	<0.05
Heilongjiang province	0.767	<0.001
Shanghai	0.712	<0.001
Jiangsu province	0.797	<0.001
Zhejiang province	0.697	<0.001
Anhui province	0.427	<0.001
Fujian province	0.400	<0.001
Jiangxi province	0.857	<0.001
Shandong province	0.491	<0.001
Henan province	0.800	<0.001
Hubei province	0.592	<0.001
Hunan province	0.711	<0.001
Guangdong province	0.810	<0.001
Guangxi zhuang autonomous region	0.201	<0.05
Hainan province	0.432	<0.001
Chongqing	0.511	<0.001
Sichuan province	0.707	<0.001
Guizhou province	0.648	<0.001
Yunnan province	0.826	<0.001
Shaanxi province	0.513	<0.001
Gansu province	0.333	<0.05
Qinghai province	0.229	<0.05
Ningxia hui autonomous region	0.168	<0.05
Xinjiang uygur autonomous region	0.261	<0.05

The incidence rate and the number of reported cases in all provinces and cities generally showed a trend of rising and then falling, which can be roughly divided into three phases in Figures 2B,C. The peak periods were mainly in late January and early February, but the situation differed from place to place. Among them, an abnormal value was observed in Hubei province due to the change of testing method on February 12th. The incidence rate and the number of reported cases were significantly higher in Hubei Province than in other regions.

### The Results of Generalized Estimation Equation

**Figure 3:**
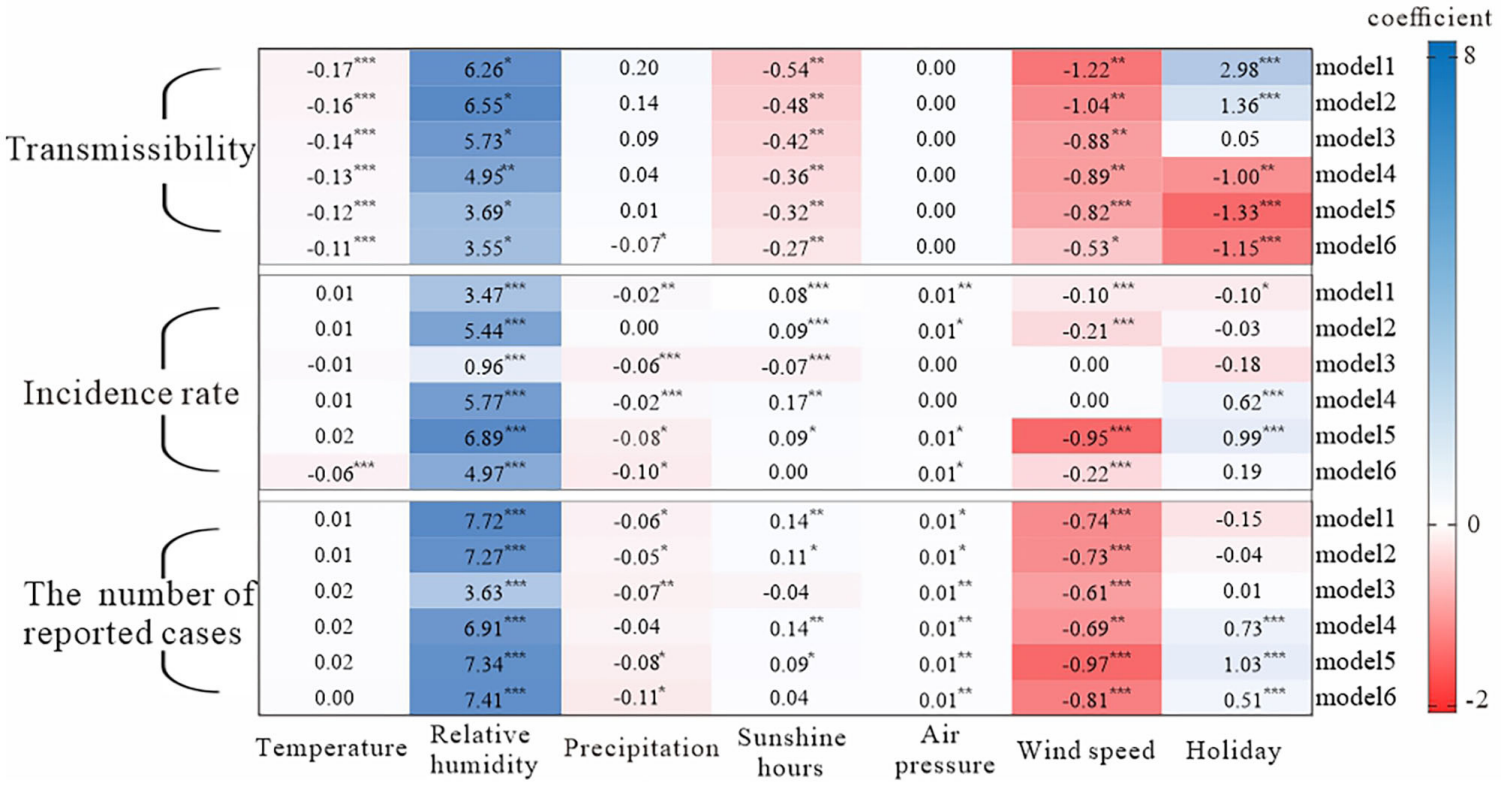
Analysis results of influencing factors based on the generalized estimation equation. Model 1: the just-in-time effect model; model 2: 1 day lag effect model; model 3: 2 days lag effect model; model 4: 3 days lag effect model; model 5: 4 days lag effect model; model 6: 5 days lag effect model; p is based on the results of the generalized estimating equation; *** = *p* < 0.001; ** = *p* < 0.01; * = *p* < 0.1. All correlation coefficients |r| were <0.8, so there was no strong correlation between x1 and x6; the variance inflation factor (VIF) values among all covariates were between 1.21 and 3.58, so the collinearity between x1 and x6 in models 1–6 was not substantial.

Figure 3 showed the results of the effects of the major meteorological factors on transmissibility, incidence rate, and number of reported cases. All correlation coefficients |*r*| were <0.8, so there was no strong correlation between the main meteorological factors. The variance inflation factor (VIF) values among all covariates were between 1.21 and 3.58, so the collinearity between the major meteorological factors in models 1–6 was not substantial. In general, temperature, relative humidity, precipitation, sunshine hours, and wind speed had immediate and lagged effects on transmissibility, with different number of days lagged. Temperature had a lagged effect on incidence rate, while relative humidity, precipitation, sunshine hours, air pressure, wind speed, and holiday had both immediate and lagged effects on both incidence rate and the number of reported cases.

We found that precipitation and wind speed had a negative effect on transmissibility, while humidity had a positive effect. The effect of temperature, sunshine hours, wind speed, and relative humidity on the transmissibility lasted for at least 5 days, and the degree of effect decreased day by day. The lagged effect of precipitation on the transmissibility lasted for 4 days. Air pressure had no effect on transmissibility. The effect of wind speed on transmissibility was the same as the effect of sunshine hours. Holiday had a negative effect on transmissibility and had a lagged effect.

The higher the temperature, the higher the incidence rate. Also, the effect of temperature on incidence rate lagged by 5 days. The remaining five meteorological factors had a timely and lagged effect on incidence rate. Precipitation had a negative effect on incidence rate, but relative humidity had a positive effect on it. Except for model 3, the longer the sunshine hours, the higher incidence rate. The results showed that air pressure had a positive effect on incidence rate, but wind speed had a negative effect. In terms of the effects of key meteorological factors on the number of reported cases, the effect was approximately the same as that on the incidence rate.

## Discussion

Overall, the SEIAR model developed in this study was able to fit the epidemic data of Chinese provinces and cities, and the fitting results were satisfactory. Based on the fitting results, the trend of transmissibility of COVID-19 in each province and city could be evaluated.

In exploring the effects of meteorological factors on the prevalence of COVID-19, different studies have used different methods and outcome variables. In a study involving the effect of meteorological factors in 429 cities, scholars defined the outcome variable as the cumulative number of cases and explored the effect of meteorological factors on the number of reported cases through the generalized linear mixed model and restricted cubic spline model (1). In another study exploring the factors influencing of the prevalence of COVID-19 in South American countries, in addition to the number of daily confirmed cases as an outcome variable, the number of daily incubation cases was defined as another outcome variable, defining the number of daily confirmed cases as the number of incubation cases 4 days earlier. These two outcome variables were analyzed separately for Spearman rank correlation with meteorological factors for each country (8). In another study on factors influencing COVID-19 in Latin America and the Caribbean, the number of new cases per day and the number of deaths were defined as outcome variables, also correlated using Spearman rank correlation (14). In addition to the number of reported cases, the incidence rate can also be used as an outcome variable. In a study exploring the effect of climatic conditions on the incidence rate of COVID-19 in 31 Chinese provinces, the daily incidence rate in each province was used as the outcome variable, and the relationship between meteorological factors and outcome variables was analyzed using locally weighted regression scatter smoothing (7). In another study comparing the impact of COVID-19 factors in Wuhan and non-Wuhan cities, daily incidence rate and daily fatality rate were used as outcome variables, respectively, and the correlation between temperature and them was analyzed by Pearson correlation or Spearman rank correlation method (4). In this study, we used daily incidence rate, the number of reported cases, and *R*_*eff*_ as outcome variables to explore the impacts of key meteorological factors on the COVID-19 transmission. Generalized estimating equations were used to calculate the immediate and lagged effects of meteorological factors on the three outcome variables.

In a large number of studies on the impact of meteorological factors on COVID-19, the results were not identical (16). In addition to the differences caused by the outcome variables and analysis methods, the treatment of meteorological factors could also influence the results. At the national level, the first-hand data available were various types of meteorological data from meteorological stations across the country. In contrast, daily meteorological data for each province were not directly available. In this study, data at the provincial level were obtained by averaging the daily meteorological data from the stations included in each province. The processing method was the same as other existing studies (1, 4, 7). Also, according to the national distribution map of weather stations, the distribution of weather stations within each province was relatively uniform. Also, the average values of the data from the stations included in each province were representative. We included the categorical variable of holiday in the generalized estimation model to control the potential confounding effects of other independent variables on the three outcome variables.

Studies had found that changes in temperature may affect the outbreak of Severe acute respiratory syndrome coronavirus (SARS) (35, 36). The survival times of Severe-Acute-Respiratory-Syndrome-Coronavirus (SARS-CoV), Middle East Respiratory Syndrome coronavirus (MERS-COV), and other coronaviruses was reduced at higher temperatures (37). In addition, lower temperatures were more conducive to the spread of influenza viruses (38). These suggested that respiratory infections like COVID-19 may also be influenced by temperature.

In a study that included 24,136 COVID-19 cases from China and 26 other countries, temperature was found to affect the cumulative number of COVID-19 cases. It was found that when the temperature rose to 30°C, the cumulative number of cases increased by only 3.38, suggesting that novel coronavirus may be highly sensitive to high temperatures (1). Similarly, it has been implied that both the number of reported cases and transmissibility of COVID-19 may be affected as the temperature continued to rise (12). Early differences in COVID-19 growth rates in different regions also reflected the effect of temperature on disease transmission (2). In a study of COVID-19 in Wuhan based on a transmission dynamic model, the *R*_0_ calculated from the model fitting was negatively correlated with temperature. The higher the temperature, the lower the transmissibility (3). In addition, studies have shown that COVID-19 mortality is also influenced by temperature (5, 39). There was evidence that mortality from respiratory diseases is affected to varying degrees by both cold and hot conditions (4). The effect of temperature on COVID-19 mortality was mostly reduced in higher temperatures in both general and severe patients (4). In this study, the effect of temperature on incidence rate and transmissibility also showed a negative correlation.

It was found that the mean positive rate of the Severe-Acute-Respiratory-Syndrome-Coronavirus-2 (SARS-CoV-2) was negatively correlated with the dose of ultraviolet radiation in the sunlight (40). The virus was rapidly inactivated by sunlight (41, 42). The results of present study were similar. The longer the sunshine hours, the lower the transmissibility. However, in this study, the sunshine duration had an opposite relationship with incidence rate and the number of reported cases, unlike other studies (43). Although sunshine affects the transmission of the virus in the external environment and influences its viral activity, it may also affect human activities. The incidence rate and the number of reported cases have been largely controlled in the provinces and cities after the implementation of various interventions, which may influence the effect of sunshine hours on the number of reported cases. Data quality and other issues may also influence the result, which need to be further studied.

It was found that the SARS virus could live for at least 5 days in the external environment when the temperature was 22–25°C and the relative humidity was 40–50%, but the survival ability of the virus decreased rapidly with the increase of relative humidity (24). The same as the influenza viruses (44). It can be seen that the viability of airborne respiratory viruses varies with the relative humidity of the environment (45). However, in the published literature, the relationship between humidity and COVID-19 was different. Some studies have found no significant correlation between absolute humidity and incidence rate (46), but more studies have shown that humidity can affect COVID-19 (5–13, 15–17, 19, 20, 22–35, 47, 48). For example, *R*_0_ and the number of daily cases were negatively correlated with humidity (3, 8). Salom et al. suggested that temperature had negative correlation with transmissibility (49). A study in Indian showed that the number of cases per day was positively correlated with relative humidity and absolute humidity (1). In this study, the influence of relative humidity was positively correlated with the three outcome variables. When the relative humidity is relatively high, there are small droplets suspended in the air, then the novel coronavirus can survive for a long time (47).

The impact of precipitation on COVID-19 also showed different results in different studies (4, 5, 9, 36, 43, 45, 48, 49). In this study, the impact of precipitation on transmission, incidence and the number of reported cases was negative, which is the same as the results of Salom et al. (49). Precipitation has been shown to significantly reduce the risk of COVID-19 (50). Besides, people avoiding going outside during rainy days may be another reason (51).

Studies have shown that wind speed affects the survival and transmission of SARS coronavirus (52), and its impact on COVID-19 has been confirmed (3, 53). In this study, higher wind speed reduced transmissibility, incidence rate, and the number of reported cases, which was the same as the results of existing studies (43). The reason may be that the virus could remain active in the air for several hours. At higher wind speed, the stability of the virus may be compromised, thus, affecting the transmission of the disease (54).

A study showed that wind speed was not correlated at all with *R*_0_ (49). Air pressure also had no effect on transmissibility in this study. Another study indicated that air pressure exhibited a statistically significant and negative impact on the COVID-19 confirmed cases (55). Therefore, the influence of air pressure on COVID-19 needs to be further explored.

In this study, we found that the effect of temperature on incidence rate was lagged. Looking at the whole transmission chain of infectious diseases, the impact of meteorological factors on the virus itself was reflected in the change of transmissibility, while the impact on the host was reflected in the change of incidence rate. On the one hand, human activity patterns and immunity would be affected by environmental factors. However, due to the unlikelihood of extreme weather and the lack of specific immunity to emerging viruses, the impact of the environment on humans during COVID-19 outbreaks is limited (7). On the other hand, environmental factors affect the virus itself more severely and rapidly than that the host, due to differences in virulence and mode of transmission of the virus in different environments (7). Therefore, it was found that the transmissibility of the virus decreasing with the increasing temperature, which then led to a decrease in the incidence rate and the number of reported cases (7). In terms of lag time, the results showed that the impact of temperature on incidence rate was lagged by 5 days, which was essentially the same as the average incubation period of COVID-19. Of course, the specific reasons for the lag effect may also be related to the micro level, and the mechanism of meteorological factors in the propagation to incidence rate process needs to be further explored. The present study also has some limitations. The number of reported cases was not fully representative of the number of new cases of COVID-19. Besides, this study did not cover many indicators of social factors to control for possible effects.

## Conclusions

In this study, the effects of meteorological factors on incidence rate and the number of reported cases were essentially the same. In contrast, there were some differences in the influence of meteorological factors on transmissibility. Precipitation and wind speed had negative effects on transmissibility, incidence rate and the number of reported cases, while relative humidity had a positive effect on them. The higher the temperature, the lower the transmissibility. Also, the effect of temperature on incidence rate was lagged, with a 5-day lag time. This may be the fact that the environmental factors affect the virus itself more severely and rapidly than the host, whereas the environment has a limited effect on humans. Thus, an increase in temperature may first cause a decrease in viral transmissibility, and then lead to a decrease in incidence rate. In addition, the mechanism of meteorological factors in the process of transmissibility to incidence rate needs to be further explored.

## Data Availability Statement

The raw data supporting the conclusions of this article will be made available by the authors, without undue reservation.

## Author Contributions

SL, JR, and FX made substantial contributions to conception and design, collected the data, and prepared Figures 1, 2. SL and JR built the model. FX, MZ, QC, BZ, YZ, ZL, BD, SY, AL, YK, and WZ run the model and analyzed the results. SL and FX wrote the manuscript. TC, YS, and Y-CC revised it critically for important intellectual content. All authors approved the final manuscript and agreed to be accountable for all aspects of the work.

## Funding

This project was partly supported by the Bill & Melinda Gates Foundation (No. INV-005834), it was also supported by National Key Research and Development Program of China (No. 2021YFC2301604), and the Scientific Research Grant of Fujian Province of China (No. Z0230104). The funders had no role in the study design, data collection and analysis, decision to publish, and preparation of the manuscript.

## Conflict of Interest

The authors declare that the research was conducted in the absence of any commercial or financial relationships that could be construed as a potential conflict of interest.

## Publisher's Note

All claims expressed in this article are solely those of the authors and do not necessarily represent those of their affiliated organizations, or those of the publisher, the editors and the reviewers. Any product that may be evaluated in this article, or claim that may be made by its manufacturer, is not guaranteed or endorsed by the publisher.

## Supplementary Material

Supplementary Figure 1 Spatial distribution map of weather stations in China.

Supplementary Figure 2 The framework of SEIAR model.
