# Supplementary figures and images for: Assessing the Impacts of Meteorological Factors on COVID-19 Pandemic Using Generalized Estimating Equations
Source: Front Public Health. 2022 Jul 1;10:920312. doi: 10.3389/fpubh.2022.920312 (PMC9284004; doi:10.3389/fpubh.2022.920312)

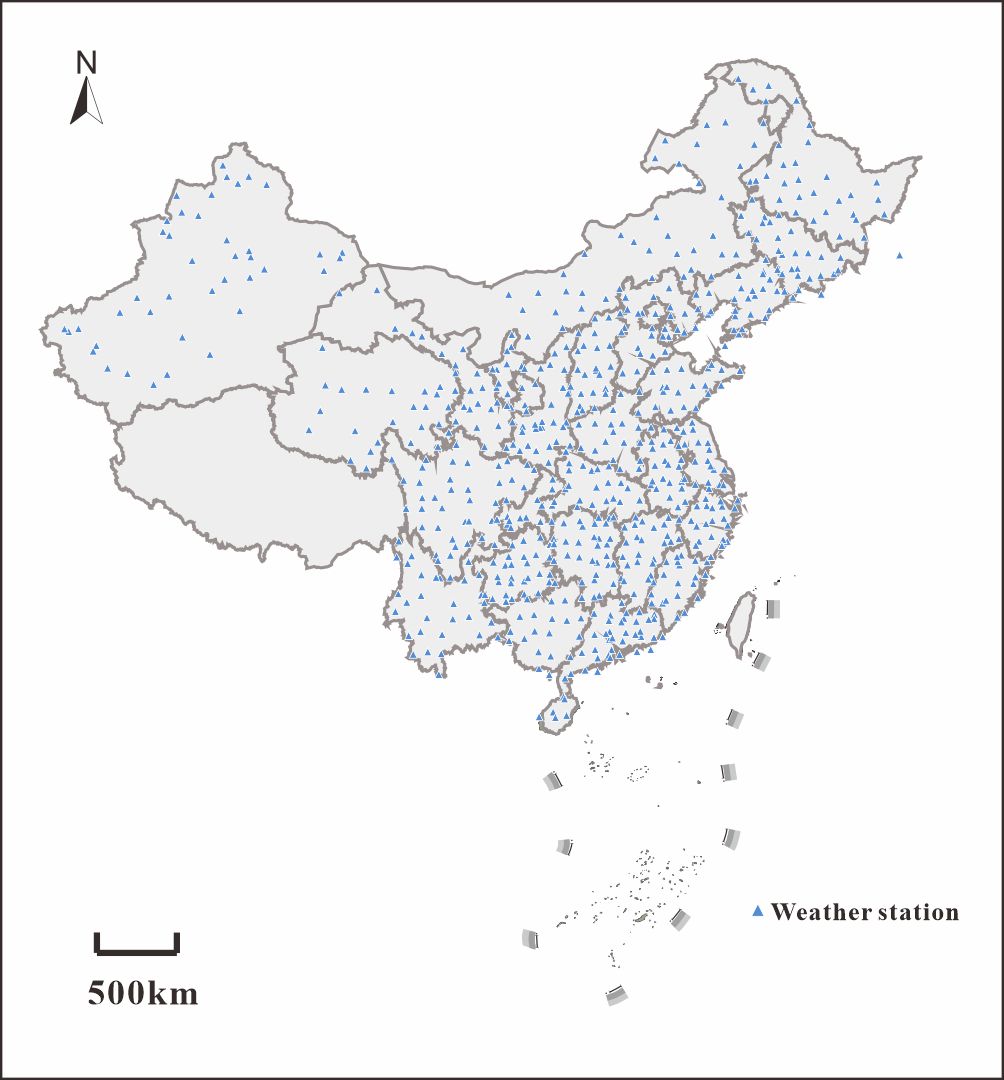

Supplement: Supplementary Figure 1 — Spatial distribution map of weather stations in China. [file Image_1.jpg]

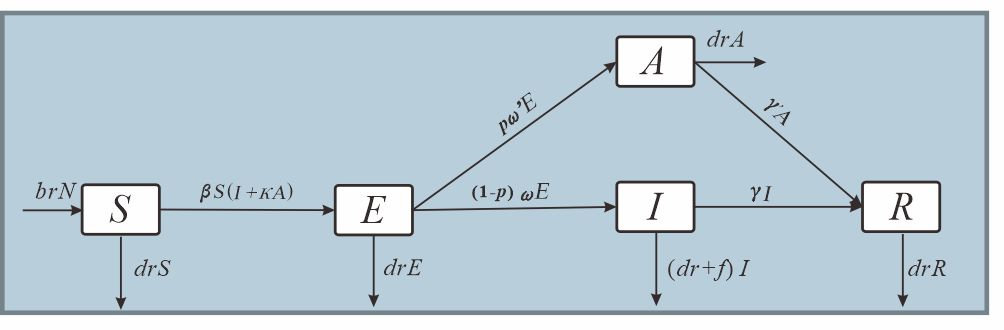

Supplement: Supplementary Figure 2 — The framework of SEIAR model. [file Image_2.jpg]
